# Single-cell transcriptomics in a child with coenzyme Q10 nephropathy: potential of single-cell RNA sequencing in pediatric kidney disease

**DOI:** 10.1007/s00467-024-06611-2

**Published:** 2025-01-14

**Authors:** Peong Gang Park, Sowon Choi, Yo Han Ahn, Seong Heon Kim, Chaeyoon Kim, Hyun Je Kim, Hee Gyung Kang

**Affiliations:** 1 Department of Pediatrics, Ajou University School of Medicine, Suwon, Republic of Korea; 2 Department of Translational Medicine, Seoul National University College of Medicine, Seoul, Republic of Korea; 3 Departments of Biomedical Sciences, Seoul National University Graduate School, Seoul, Republic of Korea; 4 Departments of Pediatrics, Seoul National University College of Medicine, Seoul, Republic of Korea; 5 Department of Microbiology and Immunology, Seoul National University College of Medicine, Seoul, Republic of Korea; 6 Genomic Medicine Institute, Seoul National University College of Medicine, Seoul, Republic of Korea; 7 Interdisciplinary Program in Artificial Intelligence (IPAI), Seoul National University, Seoul, Republic of Korea; 8 Cancer Research Institute, Seoul National University College of Medicine, Seoul, Republic of Korea

**Keywords:** Coenzyme Q10 nephropathy, *COQ2* mutation, Hereditary nephropathy, Single-cell transcriptomics, Single-cell RNA sequencing

## Abstract

**Background:**

Coenzyme Q10 (CoQ10) nephropathy is a well-known cause of hereditary steroid-resistant nephrotic syndrome, primarily impacting podocytes. This study aimed to elucidate variations in individual cell-level gene expression in CoQ10 nephropathy using single-cell transcriptomics.

**Methods:**

We conducted single-cell sequencing of a kidney biopsy specimen from a 5-year-old boy diagnosed with a CoQ10 nephropathy caused by a compound heterozygous *COQ2* mutation complicated with immune complex-mediated glomerulonephritis. The analysis focused on the proportion of cell types, differentially expressed genes in each cell type, and changes in gene expression related to mitochondrial function and oxidative phosphorylation (OXPHOS).

**Results:**

Our findings revealed a uniform downregulation of mitochondrial gene expression across various cell types in the context of these mutations. Notably, there was a specific decrease in mitochondrial gene expression across all cell types. The study also highlighted an altered immune cell population proportion attributed to the *COQ2* gene mutation. Pathway analysis indicated a downregulation in OXPHOS and an upregulation of various synthesis pathways, particularly in podocytes.

**Conclusions:**

This study improves our understanding of CoQ10 nephropathy’s pathogenesis and highlights the potential applications of single-cell sequencing in pediatric hereditary kidney diseases.

**Graphical Abstract:**

**Figure:**
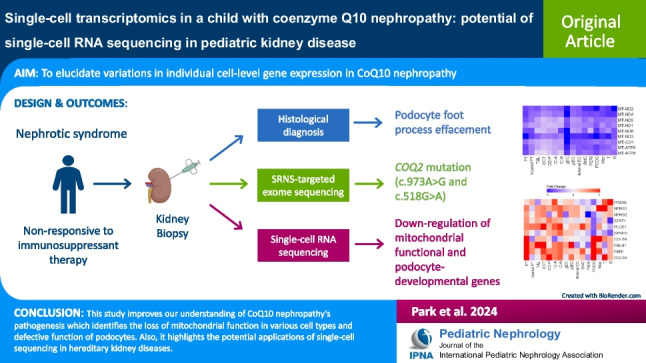
A higher resolution version of the Graphical abstract is available as Supplementary information

**Supplementary Information:**

The online version contains supplementary material available at 10.1007/s00467-024-06611-2.

## Introduction

The association of primary coenzyme Q10 (CoQ10) deficiency with human diseases was first established in 1989, based on a clinical study conducted by Ogasahara and colleagues [1]. Subsequently, in 2006, the molecular etiology of CoQ10 deficiency was elucidated with the identification of a gene mutation impairing CoQ10 biosynthesis [2]. Recent years have seen an escalation in reported cases of primary CoQ10 deficiency, characterized by a diverse array of organ involvements, including steroid-resistant nephrotic syndrome (SRNS). From the latter half of 2010, the term “CoQ10 nephropathy” began to be used to refer to inherited kidney disease, especially glomerulopathy such as SRNS, caused by autosomal recessive mutations in the genes related to the CoQ10 biosynthesis pathway. Previous research reported that CoQ10 nephropathy due to mutations in genes such as *PDSS2*, *COQ2*, *COQ6*, and *COQ8B* is identified in approximately 1–2.7% of SRNS cases [3]. Our previous research, however, revealed a higher prevalence; among 291 Korean children diagnosed with SRNS or focal segmental glomerulosclerosis, we detected mutations in 127 patients, with 20 (6.9%) of these mutations being identified as CoQ10 nephropathy [4, 5]. This finding suggests a greater frequency of these mutations than previously reported. An early diagnosis of primary CoQ10 nephropathy is essential because the condition is possibly treatable when CoQ10 supplementation is started at the early stage [6].

Single-cell RNA sequencing (scRNA-seq) has emerged as a pivotal technique for dissecting gene expression at the level of individual cells, offering unparalleled insights into the intricate and varied cellular reactions within diverse biological settings [7]. Employing scRNA-seq, novel insights have been gained into the cell type-specific characteristics of various kidney diseases. A notable example includes chronic kidney disease (CKD), where significant changes in the gene expression of proximal tubule (PT) cells have been observed [8]. However, prior research employing scRNA-seq has predominantly concentrated on prevalent and/or immunologically driven conditions, such as CKD and lupus nephritis, thereby highlighting the need for broader application of this technique across a wider spectrum of kidney disorders, such as inherited kidney diseases [9].

In this study, we identified a novel compound heterozygous variant in the *COQ2* gene of a patient with SRNS and concomitant immune-mediated glomerulonephritis and further explored variations of individual cell-level expression using single-cell transcriptomics. We compared the single-cell transcriptomic data of kidney tissue from the patient with a reference database and observed a uniform downregulation of mitochondrial gene expression across various cell types in this patient [10]. This was accompanied by an increase in glycolysis-related gene expression, specifically in the podocytes. Pathway analysis revealed a downregulation of oxidative phosphorylation (OXPHOS) and an upregulation of synthesis pathways in the podocytes. These insights contribute to our understanding of the pathogenesis and potential therapeutic approaches for primary CoQ10 nephropathy.

## Methods

### Clinical manifestations, histological analysis, and genetic analysis

We collected the patient’s clinical information, which includes demographic information, symptoms, results of laboratory tests, and the medical history of the patient’s family. The sample from the core needle biopsy was prepared for histological analysis under both light and electron microscopes. To conduct genetic analysis, we extracted genomic DNA from the blood samples of the patient and his parents and then performed whole-exome sequencing. We applied a targeted gene capture method to specifically examine 68 genes associated with conditions related to Mendelian nephrotic syndrome [11]. The pathogenicity of the detected *COQ2* mutations was assessed following the American College of Medical Genetics and Genomics guidelines [12].

### Tissue procurement, single-cell isolation, and gel bead in emulsion generation

Fresh human kidney tissue was obtained from a needle biopsy at the Seoul National University Children’s Hospital. The kidney tissue was transported on ice in media containing 10% FBS in RPMI1640 (SH30027.01; Thermo Fisher Scientific, Waltham, MA, USA) and then washed in cold PBS (ML008-01; Welgene, Gyeongsangbuk-do, Republic of Korea). The tissue was chopped and dissociated with Liberase™ TL (#05401020001; Roche, Mannheim, Germany, 0.25 mg/mL in PBS) in a 37 °C water bath for 30 min. The tissue was then mechanically dissociated using a wide-bore pipette tip. Afterward, the cells isolated from the tissue were filtered through a strainer with a mesh size of 70 µm (#93,070; SPL Life Sciences, Gyeonggi-do, Republic of Korea), followed by centrifugation (500 g, 5 min, 4 °C). Then, the cells were resuspended with 60 µL of cold media for cell counting and then diluted to the proper cell concentration. The cells were immediately loaded onto the 10X Chromium Controller.

### Library preparation and NGS sequencing

Libraries were generated using the Chromium Single Cell 3′ Library & Gel Bead Kit V3.1 and Chromium Single Cell 3′ Chips according to the manufacturer’s instructions. In short, single-cell samples and reagents were loaded on a 10X Chromium Controller for droplet generation, followed by reverse transcription in the droplets, cDNA amplification, and fragmentation. Then, fragments were ligated with adapters and dual indices to mitigate index-hopping. The 4150 TapeStation system (Agilent, Santa Clara, CA, USA) was used to evaluate the quality of the barcoded single-cell transcriptome libraries. The libraries showed a peak of around 460 bp and ranged between 300 and 700 bp, indicating they were of sufficient quality for sequencing (Supplementary Methods). After quality control, the libraries were sequenced with NovaSeq 6000.

### Single-cell RNA-seq data analysis

Raw sequencing reads were aligned to GRCh38 (human) using Cell Ranger v7.1.0. Low-quality single cells with fewer than 200 detected genes or more than 3000 detected genes were filtered out. Additionally, cells with mitochondrial genes constituting over 50% of all genes were also removed using Seurat package v5.0.1. Single-cell data from the patient and five normal adults from a public database were integrated and batch-corrected using the HarmonyIntegration method (Supplementary Methods) within the Seurat package [10]. We conducted three steps, including RunUMAP, FindNeighbors, and FindClusters functions from Seurat. FindClusters resolution parameter was adjusted to 0.4. Seurat was used to find cluster-specific genes, and then each cluster was manually annotated, referring to public kidney scRNA-seq data [13]. Differentially expressed genes (DEGs) were identified by using the FindMarkers function, including the Wilcoxon rank-sum test, and selected according to the following criteria: upregulated DEGs, average log2 fold change > 0.5, and adjusted *p*-value < 0.05 based on Bonferroni correction using all features in the dataset; downregulated DEGs, average log2 fold change < − 0.5, and adjusted *p*-value < 0.05. The complete set of DEGs is described in the Supplementary Data. Gene expression fold changes between the disease group and control group were analyzed for gene sets related to CoQ10, mitochondrial function, OXPHOS, and glycolysis across our clusters and then visualized with a heatmap. To conduct pathway enrichment analysis, gene sets associated with biological processes from the Gene Ontology in MSigDB were selected [14]. For input, we used the log2-fold change values from the Seurat DEG analysis. The most significantly enriched pathways were identified by GSEA, utilizing gene ontology: biological process term analysis, focusing on specific cell clusters, including podocytes.

## Results

### Clinical manifestation

**Fig. 1:**
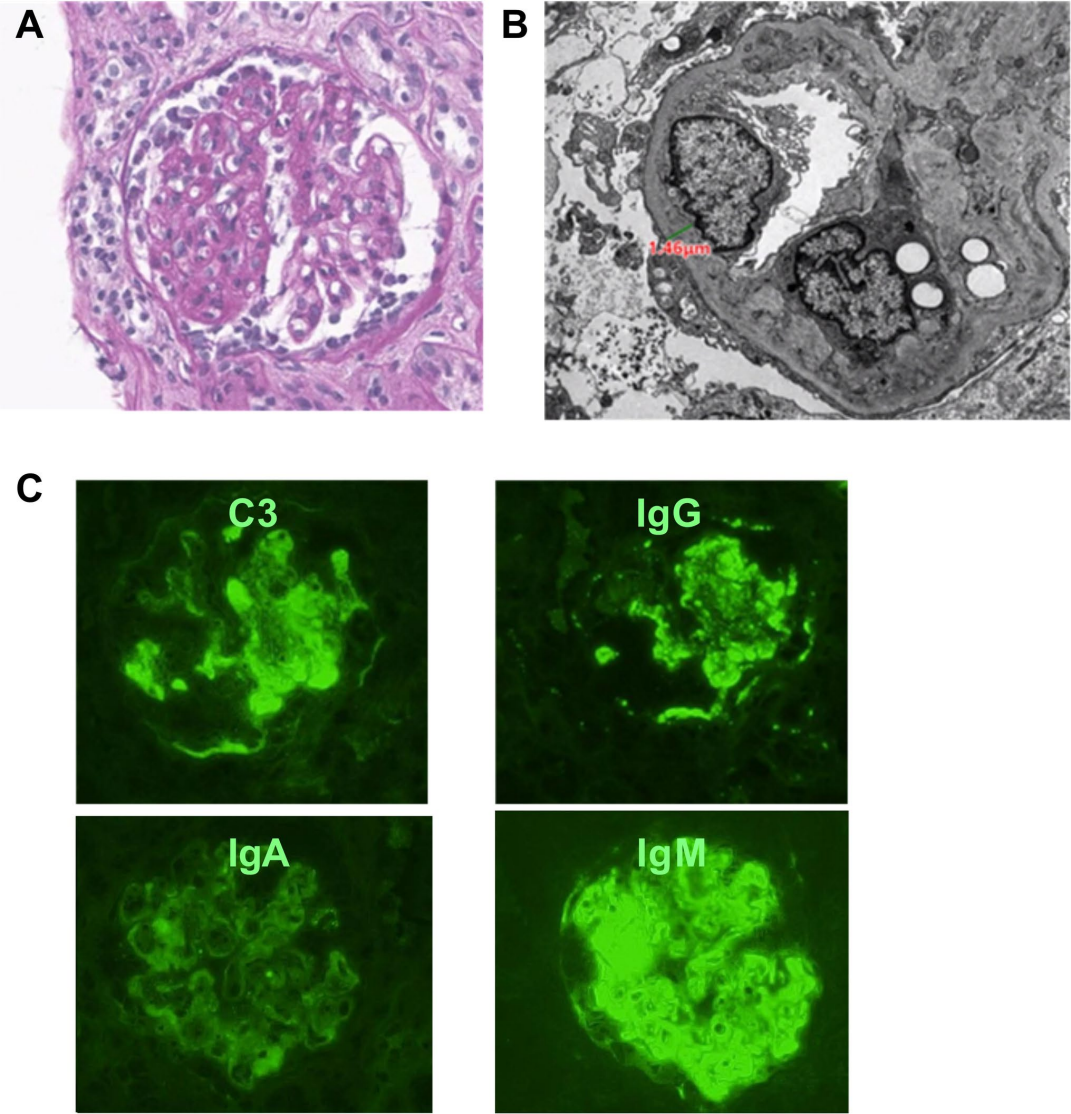
Histological findings of the patient: **A** light microscopy, **B** electron microscopy, **C** immunofluorescence staining

A 6-year-old Korean boy was transferred to our hospital due to persistent edema, having been previously healthy with normal growth and developmental milestones. Three weeks before his transfer, he was noted to have foamy urine and edema. Initial laboratory findings at the previous hospital were indicative of nephrotic syndrome (Table 1). Despite receiving standard-dose glucocorticoids for 20 days, his proteinuria persisted, and kidney function declined. A kidney biopsy revealed glomerular mesangial hyperplasia, tubulointerstitial lesions, and both global and segmental sclerosis of the glomeruli with crescent formation. Immunofluorescence microscopy showed diffuse mesangial and peripheral deposits of IgM, C3, and C1q. Electron microscopy identified small mesangial and subendothelial electron-dense deposits with foot process effacement (Fig. 1). These findings, along with the lack of response to steroid therapy and the exclusion of various secondary causes of glomerulonephritis, including infections, led to the diagnosis of immune complex membranoproliferative glomerulonephritis (IC-MPGN).

**Table 1:** Baseline patient characteristics

Parameters	Initial presentation	4 weeks after steroid	Reference range
Total protein (g/dL)	3.9	3.5	6.0–8.0
Albumin (g/dL)	1.8	1.5	3.3–5.2
Serum creatinine (mg/dL)	0.84	1.16	0.4–0.8
eGFR (mL/min/1.73 m^2^)	56.4	40	
Total cholesterol (mg/dL)	505	810	0–240
Triglyceride (mg/dL)	1060	637	0–200
C3 (mg/dL)	64	59	80–173
C4 (mg/dL)	19	19	13–46
IgG (mg/dL)	56	24	540–1822
Urine			
Protein	4 +	4 +	
RBC (/HPF)	1–4	1–4	0–4
Protein/creatinine ratio	84.1	40.4	< 0.2

*eGFR* estimated glomerular filtration rate, *RBC* red blood cell

**Fig. 2:**
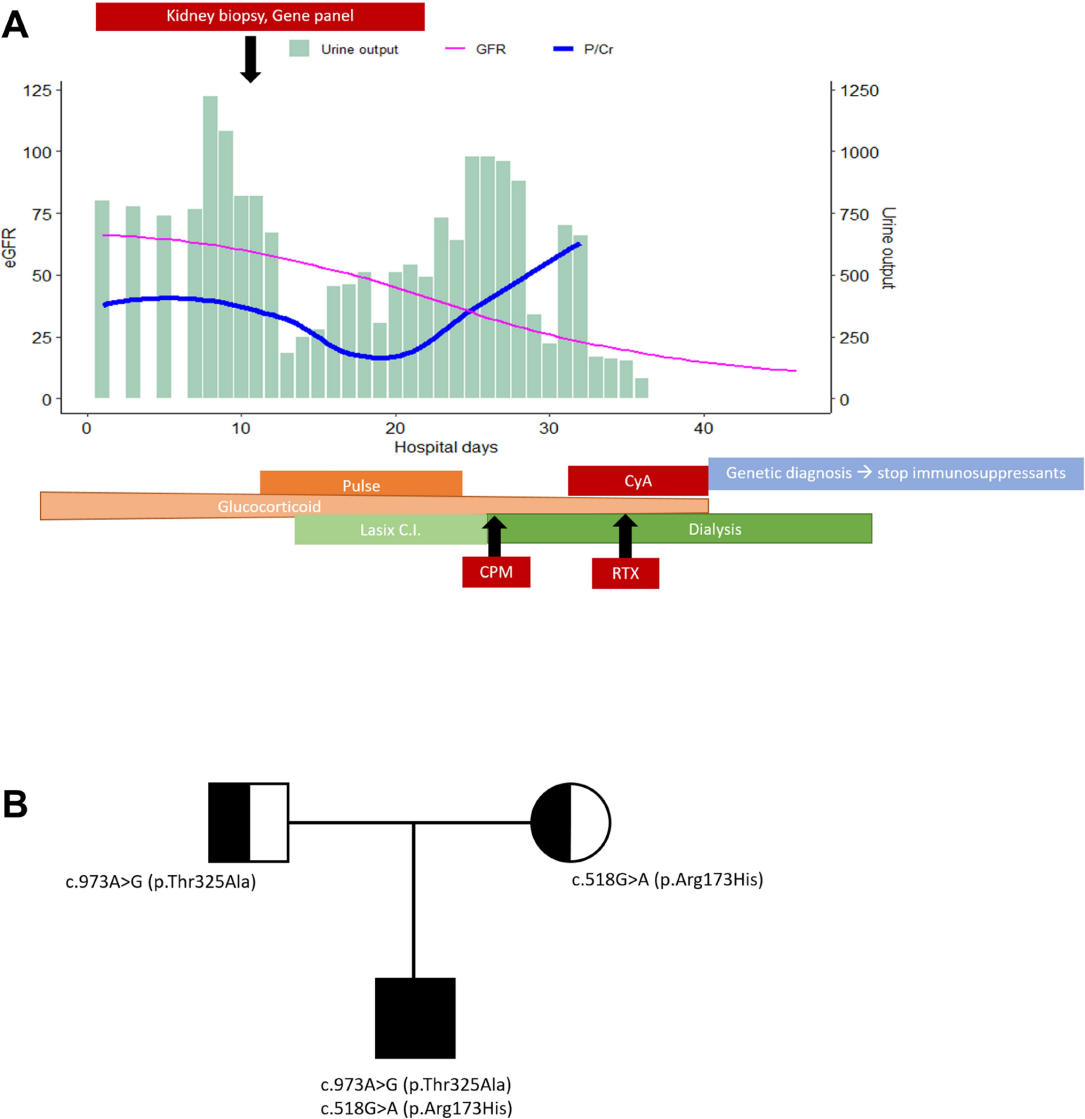
**A** This panel illustrates the patient’s clinical course. The pink line represents a gradual decline in estimated glomerular function, despite ongoing treatment. Concurrently, urine output (shown as a green histogram) diminished, accompanied by significant proteinuria (blue line). **B** Genetic analysis with a segregation study identified a compound heterozygous mutation in the *COQ2* gene. Abbreviations: eGFR, estimated glomerular filtration rate; P/Cr, urine protein/creatinine ratio; CyA, cyclosporin A; C.I., continuous infusion; CPM, cyclophosphamide; RTX, rituximab

Despite intensive immunosuppressive therapies targeting membranoproliferative glomerulonephritis, the patient’s kidney function continued to deteriorate (Fig. 2A). Targeted exome sequencing for SRNS belatedly revealed compound heterozygous likely pathogenic variants of *COQ2*, c.518G > A (p.Arg173His) from the father and c.973A > G (p.Thr325Ala) from the mother [9] (Fig. 2B). We considered that in this patient, who has a predisposing condition due to a *COQ2* mutation, severe IC-MPGN developed, and was unresponsive to various types of immunosuppression; immunosuppressive therapy was discontinued, and high-dose CoQ10 supplementation was initiated. Maintenance kidney replacement therapy was initiated 2 months after disease onset; tragically, 6 weeks after the initiation of dialysis, the patient died with cardiopulmonary failure following a fever of unknown cause.

### Single-cell transcriptome analysis

**Fig. 3:**
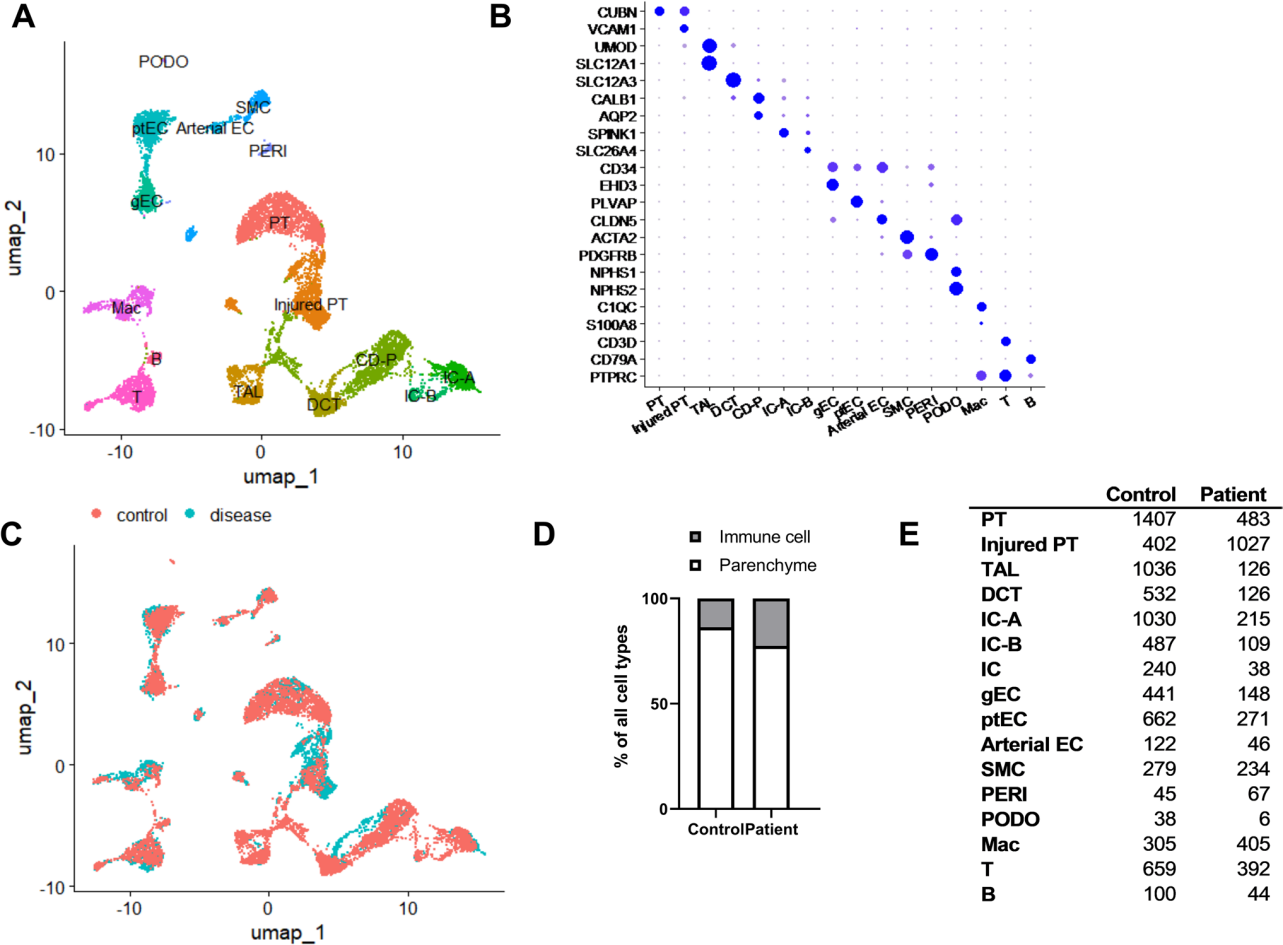
**A** Displaying 11,522 cells (3737 from the patient and 7785 from the control) post quality control and dataset integration via Harmony. The UMAP projection identifies 18 cell clusters including proximal tubule (PT), thick ascending limb (TAL), distal convoluted tubule (DCT), collecting duct principal cells (CD-P), types A and B intercalated cells (IC-A, IC-B), glomerular (gEC), and peritubular endothelial cells (ptEC), arterial endothelial cells (Arterial EC), smooth muscle cells (SMC), pericytes (PERI), podocytes (PODO), macrophages (Mac), and lymphocytes (T and B cells). **B** Cell type-specific expression of marker genes across manually annotated clusters, with dot size indicating the percentage of cells expressing each marker and color scale reflecting average gene expression levels. **C** Cells exhibit unbiased distribution in relation to disease status after batch correction. **D** The fractions of parenchymal and immune cells. **E** The number of cell types between the control and the patient

To investigate the altered gene expression and pathways in this patient at a single-cell level, we obtained a kidney biopsy sample and generated transcriptome datasets from 3737 cells after excluding low-quality cells. The patient’s single-cell transcriptomics data were integrated with transcriptomics data from five young adults (aged 20 to 40 years) with normal kidney function, sourced from the public Kidney Precision Medicine Project database [10]. Following batch correction and integration with the public dataset, we performed unsupervised clustering, resulting in 15 distinct cell types (Fig. 3A). Each cluster was annotated using canonical cell type-specific marker genes for kidney epithelial, endothelial, and immune cells (Fig. 3B). The general cellular composition was similar between the patient and the control group (Fig. 3C). However, the proportion of immune cells was higher in the patient, while the control group exhibited a greater proportion of parenchymal cells, particularly endothelial cells (Fig. 3D and E). The elevated immune cell count in the patient might contribute to the development of a membranoproliferative pattern of glomerulonephritis [15].

**Fig. 4:**
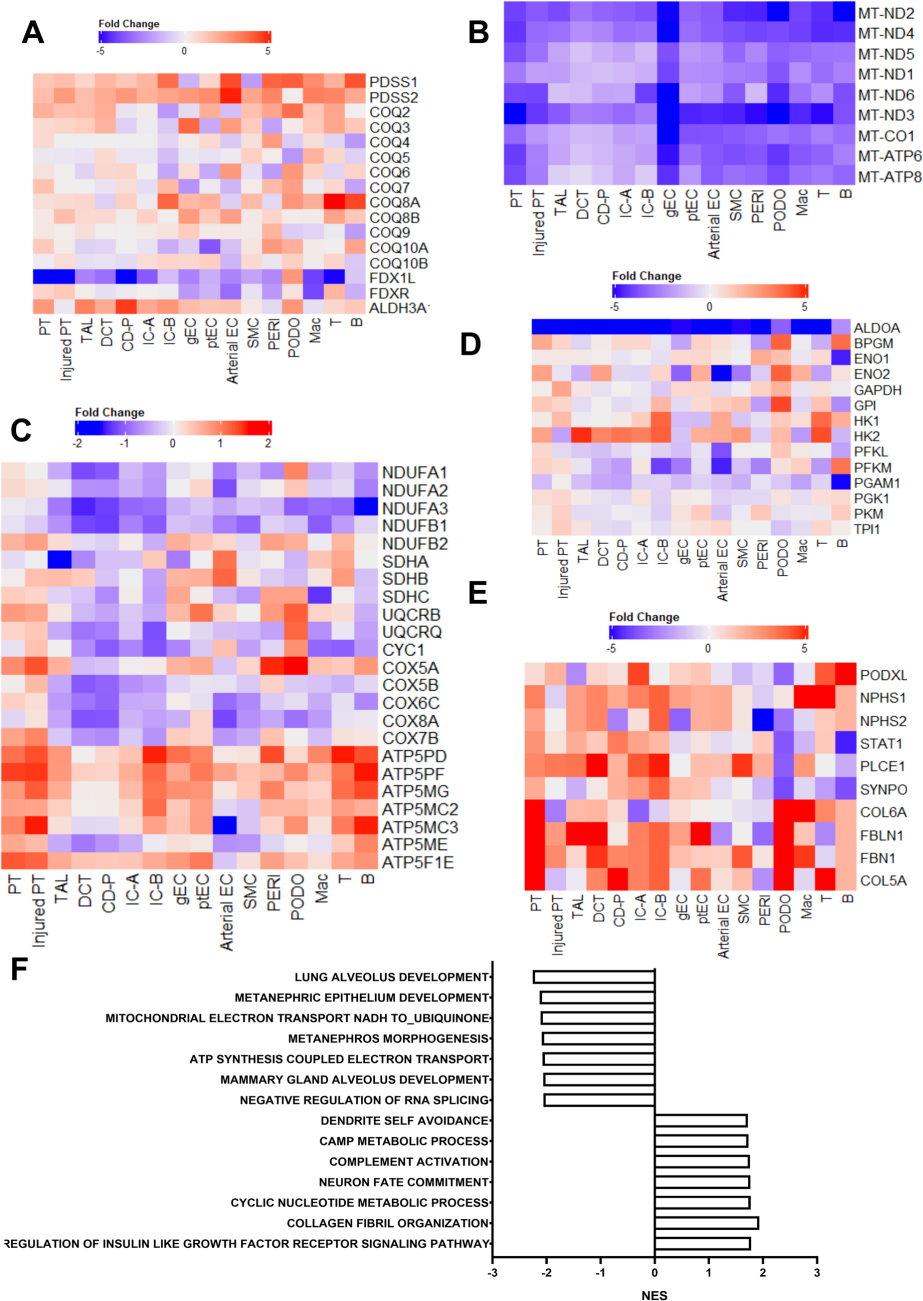
Heatmap shows the fold changes between the patient and the control of selected genes: **A** CoQ10 biosynthesis-related genes. **B** Mitochondrial genes. **C** Oxidative phosphorylation-related genes. **D** glycolysis-related genes. **E** Podocyte development and fibrosis-related genes. **F** Pathway analysis in podocytes

Given the genetic confirmation of a *COQ2* mutation in this patient, we analyzed the expression of CoQ10-related genes, including *COQ2*. As depicted in Fig. 4A, *COQ2* mRNA expression was detectable in the patient, suggesting that the mutation leads to a functional defect rather than an alteration in expression level. However, we observed a notable upregulation of *COQ6* and *COQ10A* and downregulation of *COQ4* and *COQ9*, particularly in podocytes. This suggests a varied impact on CoQ10 biosynthesis, especially in podocytes.

Further, we examined changes in the expression levels of mitochondrial genes and found a uniform downregulation across all cell types, indicating a severe impairment in mitochondrial function (Fig. 4B). We also assessed changes in genes related to OXPHOS, a key mitochondrial function. While genes associated with Complexes I, II, III, and IV were downregulated, those related to Complex V (ATP synthase) were upregulated in various cell types (Fig. 4C). Interestingly, there was a specific upregulation of glycolysis-related genes in podocytes (Fig. 4D). Notably, genes linked to podocyte development were uniquely downregulated in the patient’s podocytes, suggesting impaired podocyte function or their dedifferentiation (Fig. 4E). Lastly, pathway analysis using hallmark gene sets revealed downregulation of OXPHOS and upregulation of fibrous tissue synthesis pathways (Fig. 4F). Additionally, TAGLN associated with fibrosis and podocyte impairment was upregulated in the patient’s podocytes compared to 5 healthy controls (Supplementary Fig. 1) [16]. However, a heatmap comparing young CKD patients (aged 20 to 40 years) with healthy controls from the same database did not show the same uniform downregulation of mitochondrial genes observed in our results (Supplementary Fig. 2). This suggests that the possibility of our findings being artifacts due to batch effects or other factors is unlikely.

## Discussion

Single-cell methodologies have facilitated the observation of gene expression alterations in thousands of distinct cells within a single experiment. In acute kidney injury, scRNA-seq has led to the discovery of a new cell type derived from proximal tubules, and an increase in interferon response and the expression of chemokine receptors in various cell clusters was demonstrated in lupus nephritis [9, 17]. However, there have been few attempts to examine gene expression through scRNA-seq in human genetic kidney diseases, and to the best of our knowledge, this study is the first to present such results. In this case, a biopsy was conducted to clarify SRNS, allowing the use of a portion of the tissue for this examination [18].

Consequently, we were able to present a single-cell transcriptome analysis of this patient, whose underlying *COQ2* mutation may have predisposed him to a more severe and rapidly progressing course of IC-MPGN; the coexistence of these conditions may have synergistically contributed to the rapid deterioration of kidney function observed in this child.

One of the major findings of this study is that while there was no significant downregulation in gene expression related to CoQ10 biosynthesis in major clusters, there was a severe reduction in the expression of mitochondrial genes in every cell cluster. It is likely that the mutation observed in this patient impaired the function, rather than the expression level, of the CoQ10 biosynthesis pathway. This functional impairment is thought to have affected mitochondrial gene expression. CoQ10 plays a pivotal role in OXPHOS, a critical process for energy generation within cells. Deficiencies in the enzymes responsible for CoQ10 synthesis can lead to a marked decrease in intracellular energy production. This deficit predominantly affects tissues with high demands for mitochondrial energy, such as the brain, kidney tubules, and podocytes, where pathological changes are likely to occur because of impaired energy metabolism [19]. In many cases of mitochondriopathies, which arise from multiple mitochondrial gene defects, severe developmental delays are often observed due to brain involvement [20]. However, the patient with *COQ2* nephropathy presented in this study exhibited normal development. Furthermore, while a significant number of diseases result in severe tubulopathy, this patient demonstrated normal tubular function. These findings suggest a distinctive pathophysiological mechanism in *COQ2* nephropathy that spares certain functions impaired in other mitochondrial disorders. Brain tissue, proximal tubules, and podocytes are all energy-intensive tissues [21]. However, further research is required to elucidate how specific gene mutations selectively impact these tissues. This necessitates a deeper understanding of the differential vulnerability and pathophysiological mechanisms in these high-energy-demand tissues. Specifically, the gene expression related to Complexes I–IV, which generate the electron gradient, was predominantly diminished, while there was an upregulation in the expression of Complex V. This observation implies a possible compensatory effect and differs from the results observed in the *Adck4-* knockout mouse, where protein expression appeared to be insignificantly altered in the knockout specimen [22]. Notably, in podocytes, there was an increased expression of genes related to glycolysis, which is also thought to be a compensatory response to impaired energy production.

Previous research on the pathophysiology of CoQ10 nephropathy is limited. Widmeier and his colleagues utilized podocyte-specific, *Adck4*- and *coq6*- knockout mouse models to clarify reduced respiratory chain activity and mitochondrial potential in the CoQ10 nephropathy model [22, 23]. This study delves deeper into the pathophysiology of this genetic disorder by demonstrating defects in mitochondrial function using human tissue, although the case presented here differs from typical pure CoQ10 nephropathy. Furthermore, it highlighted the potential for employing scRNA-seq in the study of other pediatric kidney diseases.

A key strength of this study lies in its methodology of preparing tissue samples. Instead of using frozen or thawed preparations, an “on-call” preparation method was employed. This approach enabled the creation of a cDNA library within 6 h of tissue acquisition from the human body, thereby minimizing gene alterations that could potentially arise from storage processes. However, this study has certain limitations. First and foremost, it appears that the disease manifesting in this patient is not solely due to pure CoQ10 nephropathy. Although the patient did not respond to various immunosuppressants clinically and initially presented with SRNS, the rapid progression to kidney failure and continuous hypocomplementemia suggest additional underlying factors. Furthermore, the diverse immunoglobulin staining seen in immunofluorescence implies that IC-MPGN may have developed concurrently. Nevertheless, the lesions identified in the scRNA-seq analysis likely reflect significant genetic defects in this patient. Second, the control group for comparison ideally should have comprised age-matched pediatric kidneys. However, since it was based on normal adults aged 20–40, there could have been differences in gene expression related to age. Third, only one patient was used in the analysis, presenting a limitation. Nevertheless, this study underscores the potential of scRNA-seq as a powerful tool in pediatric nephrology research, particularly for rare pediatric kidney diseases. Despite the co-existence of IC-MPGN in our patient, our findings demonstrate how scRNA-seq can reveal specific gene expression patterns associated with genetic mutations, such as the downregulation of mitochondrial genes in *COQ2* nephropathy.

In conclusion, this study provides a complementary investigation of the clinical and molecular response of the kidney in CoQ10 nephropathy. Transcriptomic evidence identifies the loss of mitochondrial function in various cell types and defective functions of podocytes. In future research, the application of scRNA-seq together with spatial transcriptomics in hereditary kidney diseases such as Alport syndrome and autosomal dominant polycystic kidney disease, which pose significant disease burdens, is anticipated to substantially broaden our understanding of their pathophysiology.

## Supplementary Information

Below is the link to the electronic supplementary material.

Graphical abstract (PPTX 1129 KB)

Supplementary file2. List of differentially expressed genes between our patient and controls, categorized by each cluster (XLSX 7181 KB)

Supplementary file3 (PDF 128 KB)

## Data Availability

The dataset for single-cell RNA transcriptomics has been deposited in the Gene Expression Omnibus database (accession no. GSE270701). This study does not report any original code, and the codes used are available in the “Methods” section. Any additional information required to reanalyze the data is available from the lead contact upon request.
