# Supplementary material for: Single-cell transcriptomics in a child with coenzyme Q10 nephropathy: potential of single-cell RNA sequencing in pediatric kidney disease
Source: Pediatr Nephrol. 2025 Jan 14;40(5):1653–62. doi: 10.1007/s00467-024-06611-2 (PMC11946986; doi:10.1007/s00467-024-06611-2)
Supplement: Supplementary file 1 — Graphical abstract (PPTX 1129 KB) [file 467_2024_6611_MOESM1_ESM.pptx]

## Slide 1
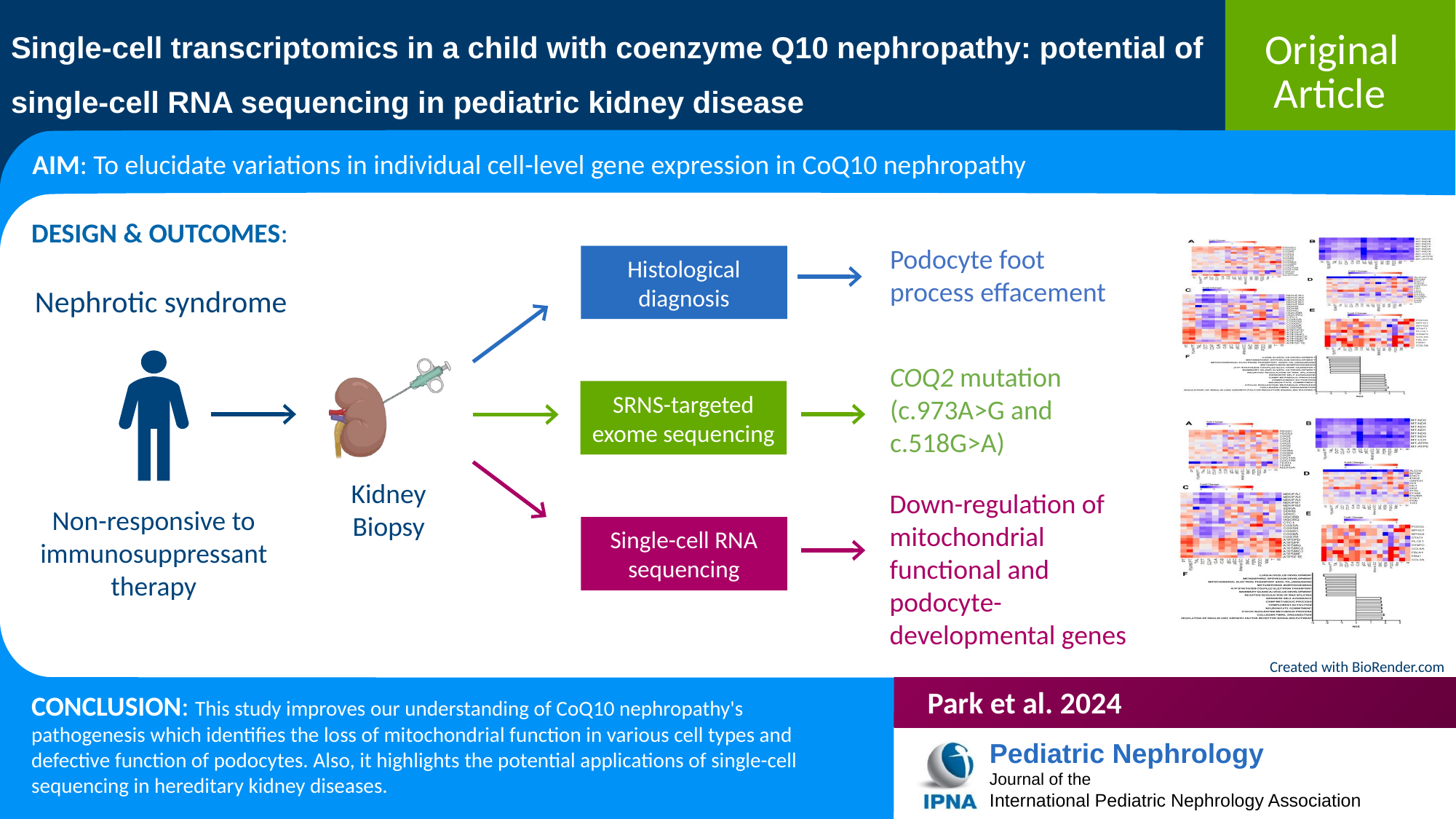

Single-cell transcriptomics in a child with coenzyme Q10 nephropathy: potential of single-cell RNA sequencing in pediatric kidney disease
AIM: To elucidate variations in individual cell-level gene expression in CoQ10 nephropathy
DESIGN & OUTCOMES:
Podocyte foot process effacement
Histological diagnosis
Nephrotic syndrome
COQ2 mutation (c.973A>G and c.518G>A)
SRNS-targeted exome sequencing
Kidney Biopsy
Down-regulation of mitochondrial functional and podocyte-developmental genes
Non-responsive to immunosuppressant therapy
Single-cell RNA sequencing
Created with BioRender.com
Park et al. 2024
CONCLUSION: This study improves our understanding of CoQ10 nephropathy's pathogenesis which identifies the loss of mitochondrial function in various cell types and defective function of podocytes. Also, it highlights the potential applications of single-cell sequencing in hereditary kidney diseases.
