# Supplementary material for: Single-cell transcriptomics in a child with coenzyme Q10 nephropathy: potential of single-cell RNA sequencing in pediatric kidney disease
Source: Pediatr Nephrol. 2025 Jan 14;40(5):1653–62. doi: 10.1007/s00467-024-06611-2 (PMC11946986; doi:10.1007/s00467-024-06611-2)
Supplement: Supplementary file 3 — Supplementary file3 (PDF 128 KB) [file 467_2024_6611_MOESM3_ESM.pdf]

Supplementary Figures

Supplementary Figure 1. Volcano plot of differentially expressed genes (DEGs) in podocytes compared between the patient (n=1) and 5 healthy references (20 – 40 years old) from Kidney Precision Medicine Project database. Significant DEGs, shown as red dots, are defined by an adjusted p-value < 0.05 and a log2 fold change > 0.5.

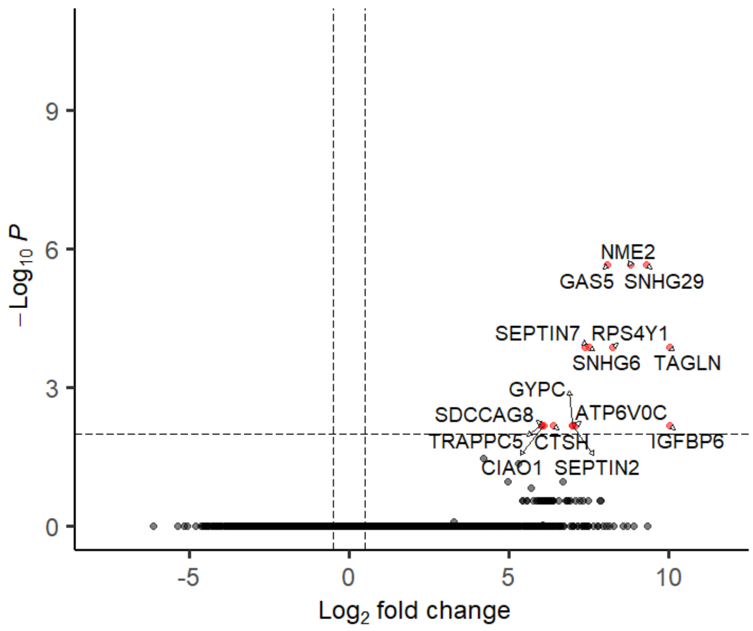

Supplementary figure 2. Heatmap of mitochondrial gene expression in 3 chronic kidney disease patients and 5 healthy controls from the Kidney Precision Medicine Project database.

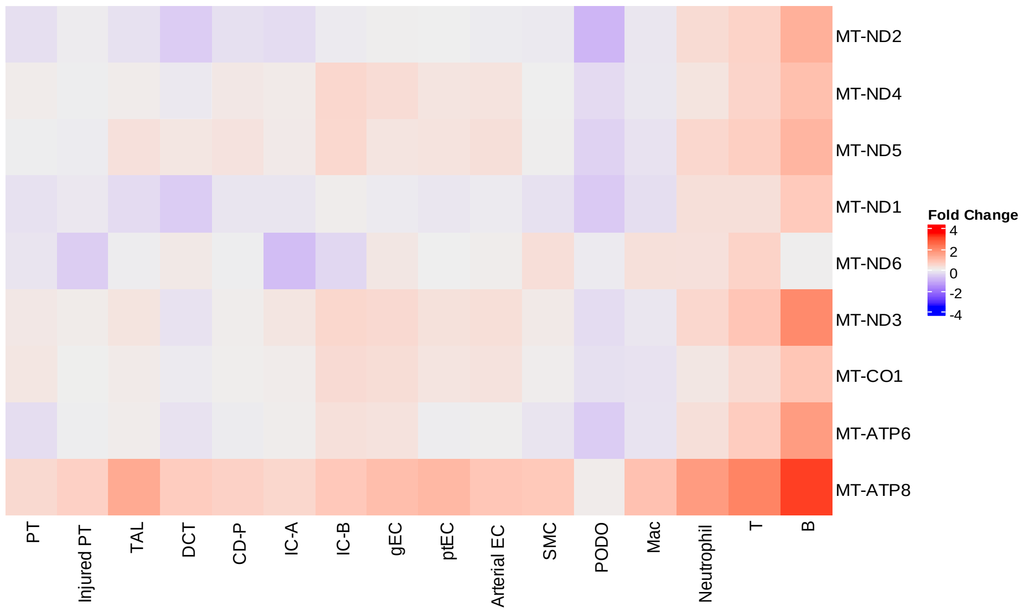

Supplementary methods

Library Quality Control

In constructing libraries, double-sided size selection was performed after the sample index PCR. This method excludes libraries longer than approximately 900 bp and shorter than 300 bp by adjusting the volume ratio between the SPRIselect beads and the sample. The libraries showed a peak around 460 bp and ranged between 300 bp and 700 bp, indicating they were of sufficient quality for sequencing.

Batch effect correction

These data were integrated using the "merge" function from Seurat. The merged control object from five normal adult datasets is labeled as "Control," and the patient data from our scRNA-seq is labeled as "Disease." HarmonyIntegration corrects batch effects through the following process. It performs dimensional reduction based on PCA, capturing the major variations in the dataset, which reflect both batch effects and biological signals in the reduced dimensional space. If identical cell types are separated and projected in different locations, this separation is considered a batch-induced variation. Following dimensional reduction, adaptive learning is applied to remove batch-specific variation and read just the cell clustering so that it is no longer determined by batch. This process is repeated to ensure that batch-specific variation does not overlap with biological signals. The key is to form clusters based on cell similarity, rather than by batch.
